# Biological Functions of Thyroid Hormone in Placenta

**DOI:** 10.3390/ijms16024161

**Published:** 2015-02-16

**Authors:** Cheng-Yi Chen, Chie-Pein Chen, Kwang-Huei Lin

**Affiliations:** 1 Department of Medical Research, Mackay Memorial Hospital, Taipei 251, Taiwan; E-Mails: csmc8972@hotmail.com (C.-Y.C.); cpchen@mmh.org.tw (C.-P.C.); 2 Division of High Risk Pregnancy, Mackay Memorial Hospital, Taipei 104, Taiwan; 3 Department of Biochemistry, School of Medicine, Chang-Gung University, Taoyuan 333, Taiwan

**Keywords:** thyroid hormone receptor, infection, inflammasome, proteomics, placenta

## Abstract

The thyroid hormone, 3,3,5-triiodo-l-thyronine (T_3_), modulates several physiological processes, including cellular growth, differentiation, metabolism, inflammation and proliferation, via interactions with thyroid hormone response elements (TREs) in the regulatory regions of target genes. Infection and inflammation are critical processes in placental development and pregnancy-related diseases. In particular, infection is the leading cause of neonatal mortality and morbidity worldwide. However, to date, no successful approach has been developed for the effective diagnosis of infection in preterm infants. Pre-eclampsia (PE) is a serious disorder that adversely affects ~5% of human pregnancies. Recent studies identified a multiprotein complex, the inflammasome, including the Nod-like receptor (NLR) family of cytosolic pattern recognition receptors, the adaptor protein apoptosis-associated speck-like protein containing a caspase recruitment domain (ASC) and caspase-1, which plays a vital role in the placenta. The thyroid hormone modulates inflammation processes and is additionally implicated in placental development and disease. Therefore, elucidation of thyroid hormone receptor-regulated inflammation-related molecules, and their underlying mechanisms in placenta, should facilitate the identification of novel predictive and therapeutic targets for placental disorders. This review provides a detailed summary of current knowledge with respect to identification of useful biomarkers and their physiological significance in placenta.

## 1. Introduction

Pioneering proteomic approaches have been continually developed over several decades. Numerous technologies aimed at investigating protein-level regulation have been established, including one-dimensional SDS-PAGE, two-dimensional gel-electrophoresis, differential in-gel electrophoresis (DIGE), isotope-coded affinity tags (ICAT), isobaric tags for relative and absolute quantification (iTRAQ), and stable isotope labeling with amino acid in cell culture (SILAC) [1,2]. Recently, the global proteomics approach was successfully applied to the systemic investigation of the regulatory proteins of tumor-associated genes [3,4,5], biomarkers for pregnancy and inflammation-associated factors [6,7,8]. Furthermore, quantitative proteomic approaches have been utilized extensively in various biological fields, such as cancer biology, protein profiling, biomarker identification and quantification, and inflammasome regulation [9,10,11,12].

Transthyretin identified as a thyroid hormone-binding protein was discovered in human cerebrospinal fluid and serum [13]. Schreiber *et al.* [14] demonstrated that transthyretin is a typical negative acute-phase plasma protein, and the expression level was decreased following trauma, surgery and inflammation in liver and blood. Dickson *et al.* [14] proposed that when the body is in trauma or under inflammatory conditions, the transthyretin transcription rates in the choroid plexus would protect the brain against hypothyroidism. Transthyretin synthesis has been identified in various organs such as liver, choroid plexus of brain and placenta. Transthyretin synthesis is implicated in transporting thyroid hormones (THs) in the brain, and brain development depends on the effects of THs [15]. Furthermore, Roef *et al.* [16] reported the ratio of free T3-to-free T4 is positively associated with the adiposity-related inflammation markers interleukin-6 and C-reactive protein, and concluded that the higher ratio of free T3 to free T4 is consistently associated with numerous markers of adverse metabolic profile and cardiovascular risk. Therefore, we speculate that the thyroid hormone may play a role in inflammation processes. Here, we provide a detailed overview of the known regulatory processes of inflammation-related proteins and their physiological significance.

## 2. The Relationship between Inflammatory Processes and Placental Disorders

The immune system plays a crucial role in regulating the processes of both term and preterm labor characterized by altering pro-inflammatory cytokines significantly in gestational tissues [17,18]. Romero *et al.* [19] displayed several factors such as uteroplacental ischemia, cervical disease, decidual hemorrhage, stress, infection, and inflammation involved in the initiation of prematurity. Labor-associated inflammatory cytokines are characterized by immunocyte infiltration and the expression of tumor necrosis factor (TNF)-α, interleukin (IL)-1β, IL-6, IL-8, and monocyte chemoattractant protein (MCP)-1 in the fetal membranes, cervix, amniotic fluid, and placenta are elevated. Expression of pro-inflammatory mediators resulted in (i) increases in prostaglandins, which promote uterine contractility; (ii) degradation of the chorioamnion extracellular matrix; and (iii) ripening of the cervix matrix metalloproteinases and reduced expression of tissue metalloproteinase inhibitors [18]. The magnitude of inflammation identified in term labor is commonly thought to be lower than in preterm labor [20]. Goldenberg *et al.* [21] suggested that the innate immune system plays an important role to trigger pro-inflammatory signaling in response to injury or infection in the premature initiation of labor. Infection is most commonly identified in the earliest preterm labors, those occurring before 28 weeks of gestation. Goldenberg and colleagues reported that 25%–40% of preterm deliveries resulted from intrauterine infection [21].

Previously, scientists have clarified that pre-eclampsia is a placental pathology with insufficient uteroplacental circulation leading to placental oxidative stress, hypoxia and infarction [22,23]. Redman *et al.* [24] proposed that pre-eclampsia develops when systemic inflammation, common to all pregnant women in the second half of pregnancy, cause one or other maternal systems to decompensate. Pre-eclampsia exhibits many classical features of inflammation and these are striking in late gestation [24,25]. Several groups have demonstrated a systemic maternal inflammatory response occurs in pre-eclampsia to affect circulating leukocytes. The granulocytes, monocytes and lymphocytes all displayed some degree of activation in pre-eclampsia compared with non-pregnant individuals [25,26]. Additionally, several scientists reported circulating pro-inflammatory cytokines are involved in pre-eclampsia. The circulating levels of TNF-α, IL-6 and IL-8 are significantly elevated in blood [27,28,29,30]. Furthermore, Xie *et al.* [31] reported that expression levels of TLR2, TLR4, IL-1β and NLRP3 are elevated in neutrophils of women with pre-eclampsia compared with normal pregnant women [32]. Recently, the NLRP3 inflammasome was demonstrated to be involved in placenta dysfunction in antiphospholipid syndrome-induced pre-eclampsia . According to these studies, we suggest that preterm labor and pre-eclampsia are inflammatory-associated disorders, and thus discuss these issues here.

## 3. Nuclear Receptor Family

The nuclear receptor (NR) superfamily is a highly conserved and extensively expressed group of ligand-mediated transcription factors in metazoans [33,34]. To date, numerous NRs have been identified in humans and mice, including classical, orphan and adopted orphan receptors [35]. Almost all NRs contain two highly conserved domains: a central DNA-binding domain and ligand-binding domain positioned along the *C*-terminus [36,37]. NRs are activated upon binding to their ligands or undergo ligand-independent activation via post-translational modifications, such as phosphorylation. These receptors mediate specific gene expression by binding to DNA or recruit coregulatory factors to influence various biological processes, including reproduction, metabolism and homeostasis. NRs have been shown to play critical roles, not only in normal homeostatic and metabolic processes, but also diseases, such as inflammation, cancer and obesity [38,39]. Furthermore, Liu *et al.* [40] used DNA constructs of hormone response elements and mass spectrometry to profile the DNA binding activity of the NR superfamily in mouse liver. Consequently, 35 members of the NR family were detected at the protein level in this study. This approach provides a useful tool to increase coverage of the whole NR proteome for application in studies on DNA binding, cellular translocation and other physiological and pathological conditions, such as inflammation, pregnancy and cancer processes [40].

## 4. Thyroid Hormone Involvement in Inflammation Processes

**Figure 1:**
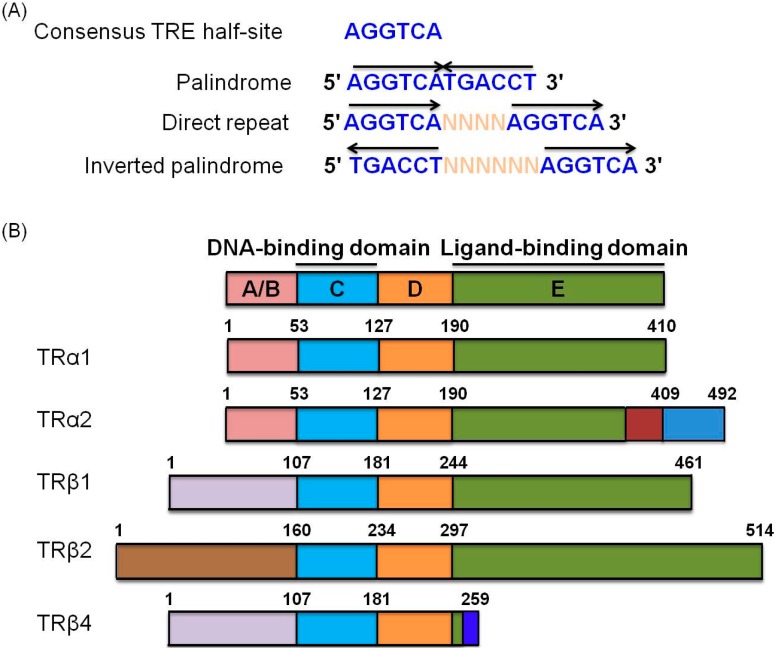
Schematic representation of consensus TRE half-sites and TRα and TRβ isoforms. (**A**) The consensus half-site of TRE is divided into palindrome, direct repeat and inverted palindrome sequences. Each half-site presents different orientations and nucleotide spacing (N: nucleotide; arrows: half-site orientation); (**B**) TRα1 and TRβ1 are generated by alternative splicing and promoter usage from TRα and TRβ. TR isoforms contain several functional domains, including an amino terminal region (A/B domain), conserved DNA-binding domain (DBD) (C domain), hinge region that links the DBD and ligand-binding domains (LBD) (D domain), and LBD responsible for receptor dimerization (E domain). Functional domains with similar amino acid sequences are depicted in the same color.

Thyroid hormone receptors (TRs) belong to the superfamily of nuclear hormone receptors that modulate transcription of various genes [41,42]. TH acts as a pleiotropic regulator of growth, differentiation, proliferation and other physiological processes through interactions with thyroid hormone response elements (TREs) located in the regulatory regions of target genes [43] (Figure 1A). Previously, Chang *et al.* [44] reported that the putative consensus hexamer half-site sequence of TRE is AGGTCA. TREs are arranged as direct repeats (DR), palindromes and inverted palindromes (IP), and vary considerably in terms of nucleotide sequence as well as spacing, number and orientation of half-sites [41,44]. Importantly, TH is required to maintain the metabolic rate and oxygen consumption in almost all tissues [37]. TRs contain several domains analogous to other nuclear receptors, including the amino terminal A/B domain, DNA-binding domain (DBD), hinge region containing the nuclear localization signal, and a carboxy-terminal ligand-binding domain (LBD) with specific functions [45,46,47,48] (Figure 1B). These receptors interact with the retinoid X receptor (RXR) to form heterodimers that influence expression of target genes by binding to their TRE regions [49]. Numerous genes, including coagulation factor system components [50], plasma proteins [50,51], nuclear receptor coactivator [52], anti-metastatic proteins [53], proteases [49] oncogenes [5] and inflammatory-associated genes [54], are regulated by thyroid hormone receptors. Proteomic approaches have identified several coregulators that interact with the thyroid hormone receptor, such as steroid receptor coactivator 1–3, thyroid hormone receptor-associated protein 220, Peroxisome Proliferator-activated Receptor-γ Coativator-1, Thyroid Hormone Receptor-binding Protein, p300, Androgen Receptor Activator 70, receptor interacting protein 140, dosage-sensitive sex reversal-adrenal hypoplasia congenital critical region of the X chromosome 1, small heterodimer partner, nuclear receptor corepressor and silencing mediator of retinoid and thyroid hormone receptors [55].

Thyroid hormone levels are associated with major physiological regulatory processes during nutritional adaptation. Recent studies have reported that leptin signaling, mediated by the JAK/STAT pathway, plays a vital role in maintaining TRH, TSH and thyroid hormone expression [56]. Obesity, enhanced by nutrient excess, is characterized by enlarged adipose cells and chronic inflammation [57]. Moreover, hyperleptinemia is reported to increase the index of autoimmune disease via regulation of proinflammatory cytokines and macrophages [58]. Thyroid hormones were linked with obesity more than a century ago, and obesity plays a critical factor in hypothyroidism [59]. Duntas *et al.* [59] reported that thyroid autoimmunity is highly related with the regulation of inflammasome-related cytokines in obesity [59]. Based on the above evidence, we speculate that inflammation may be regulated through interactions between coregulators and the thyroid hormone receptor.

## 5. Thyroid Hormone Is Associated with Placental Development and Disease

Abnormalities of the maternal thyroid hormone in pregnancy are associated with neurodevelopmental deficiencies in offspring [60,61]. Additionally, T_3_ and T_4_ exert effects in several fetal organs, including limbs, brain and liver, from 6–12 weeks of gestation [62]. TH receptors, TH transporters and deiodinase enzymes are expressed in the fetal cerebral cortex at seven weeks gestation. Hence, maternal THs play an essential role in early gestation [63,64]. Maternal T_4_ crosses the placenta in the first and third trimester of pregnancy. Clinical studies have shown that decreased TH levels can result in serious adverse effects on intellectual development of fetus [65,66,67]. Maternally-derived TH, when transported through the placenta, influences neural progenitor proliferation, migration and differentiation within the developing embryo before the onset of endogenous TH production [68,69]. Oki *et al.* [70] reported the effects of maternal thyroid dysfunction on placental development. Furthermore, THs have been shown to influence villous and extravillous trophoblast proliferation, invasion and viability *in vitro* [71]. Larijian *et al.* [72] reported that free T4 and TSH levels were increased significantly in patients with mild pre-eclampsia compared with healthy controls. Furthermore, in severe pre-eclampsia cases, the TSH value was higher, but free T3 and free T4 were significantly lower than in controls. The evidence provided a hint that hypofunctioning of the thyroid can result in mild pre-eclampsia and possibly contribute to the pathogenesis. Kumar *et al.* [73] reported that TSH levels were significantly higher in pre-eclampsia patients compared with controls. However, thyroid hormones values were in the normal range. The TSH titers were over 5 mIU/mL in about 40% pre-eclamptic patients in the study group compared with 12.2% in the controls. Additionally, the T3, free T3 and free T4 levels were lower in preterm infants born to pre-eclamptic mothers with placental insufficiency than preterm infants born to mothers without placental insufficiency. However, the TSH value was higher in preterm infants born to pre-eclamptic mothers with placental insufficiency. Hence, high levels of TSH and low levels of thyroid hormones in cord blood of premature infants born to pre-eclamptic mothers with placental insufficiency imply intrauterine hypothyroidism [73]. Kurlak and co-workers similarly reported lower thyroid hormone levels in pre-eclampsia pregnancy cases [74]. Collectively, we speculate that the levels of thyroid hormone or TSH or hypothyroidism may be a factor to influence pre-eclampsia.

## 6. Inflammasome Functions in Placental Disease

The innate immune system is activated under conditions of microbial infection, such as pathogen-associated molecular patterns (PAMP), damage-associated molecular patterns (DAMP), and tissue damage. Macrophages are vital players in the innate defense system. Activated macrophages secrete proteins to influence the immune response and recruit other immune cells to the tissue damaged area of infected sites [75]. Valimaki *et al.* [76] analyzed the human primary macrophage secretome stimulated with monosodium urate (MSU) using the high-throughput quantitative gel electrophoresis liquid chromatography-mass spectrometry/mass spectrometry (GeLC-MS/MS) approach combining protein separation via SDS-PAGE and identification with liquid chromatography-MS/MS. MSU, a metabolite released by uric acid, acts as an endogenous DAMP molecule [77]. Results based on bioinformatic analyses clearly indicate that MSU stimulates protein secretion via an unconventional mechanism. Several proteins within the secretome are secreted via vesicle-mediated processes, including the pro-inflammatory cytokines IL-18, IL-1β, interferon-induced proteins and danger signal proteins. Additionally, active forms of lysosomal proteases belonging to the cathepsin family are secreted after MSU stimulation, and cathepsin activity is essential for MSU-induced unconventional protein secretion. MSU mediates protein secretion via the cathepsin-src-Pyk2-PI3 kinase signal cascade in human macrophages [76]. Cysteinyl aspartate-specific proteases (caspases) play critical roles in inflammation and apoptosis [78]. In humans, 11 members of the caspase family have been identified, which are divided into three phylogenetic groups depending on their function [79]. Aspartate-specific cysteine protease caspase-1 is activated in the inflammasome-mediated inflammation process, and responsible for the maturation and release of the cytokines IL-1β and IL-18 during infection and inflammation [80]. Lamkaanfi and co-workers applied a proteome-wide gel-free differential peptide sorting methodology to identify novel caspase-1 substrates. Among the 1022 proteins identified, 20 were validated as caspase-1 substrates for the first time. The results additionally revealed the existence of a nucleotide binding and oligomerization domain-like receptor/caspase-1/caspase-7 cascade in response to microbial stimuli and bacterial infection [81].

**Figure 2:**
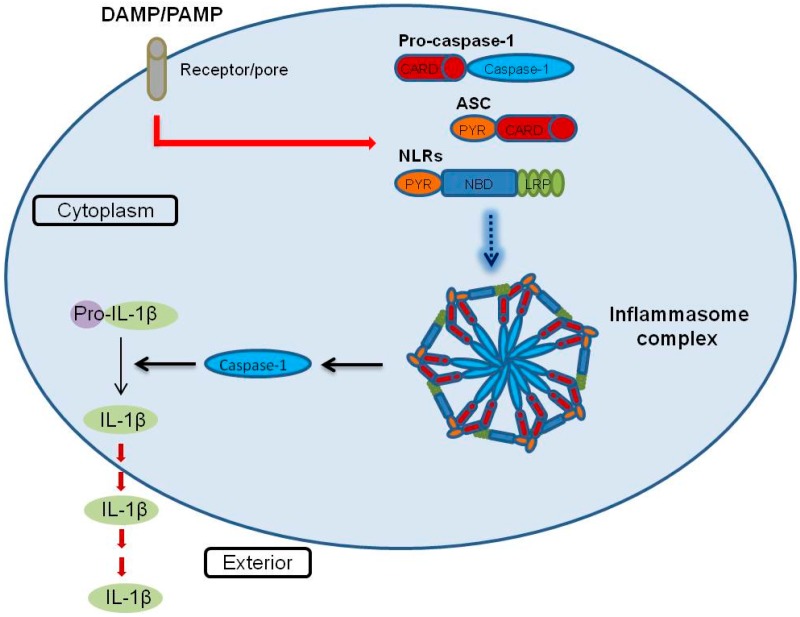
Schematic representation of the inflammasome activation model. Model depicting the regulation of inflammasome activation. Foreign factors, such as DAMP and PAMP, stimulate NLRs, ASC and pro-caspase-1, followed by formation of the inflammasome complex. Subsequently, pro-caspase-1 is cleaved into caspase-1 via activation of the inflammasome complex. Pro-IL-1β is cleaved into mature IL-1β and secreted to the macrophage extracellular matrix. (DAMP: damage-associated molecular patterns, PAMP: pathogen-associated molecular patterns, NLR: Nod-like receptor, ASC: apoptosis-associated speck-like protein containing a caspase recruitment domain, IL-1β: interleukin-1β).

Proteases are distributed extensively, constituting more than 2% of the total human genome, and play diverse roles in cellular processes, such as cell death, catabolism and immune function [82,83]. Human caspases (cysteine aspartyl proteases) consist of 12 homodimeric cysteine proteases that cleave various proteins. The human caspase family is divided into three categories based on their sequences and functional similarities. Caspase-1, -4, and -5 are inflammatory caspases, identified as activators of proinflammatory cytokines [84]. Although caspase-1 is known to be involved in the inflammasome mechanism, the underlying mechanisms by which inflammatory caspases exert their effects are yet to be elucidated [85]. The inflammasome has been recently defined as a multiprotein complex including a Nod-like receptor (NLR) family of cytosolic pattern recognition receptors, the adaptor protein apoptosis-associated speck-like protein containing a caspase recruitment domain (ASC), and caspase-1 [86]. Inflammasomes are activated by damage-associated molecular patterns (DAMPs) and pathogen-associated molecular patterns (PAMPs), such as bacterial toxins, viral DNA and RNA, extracellular ATP and reactive oxygen species (ROS) [87,88,89,90]. Inflammasome assembly leads to caspase-1-dependent cleavage and release of proinflammatory cytokines, IL-1β and IL-18 [91] (Figure 2). The substrates of these inflammatory caspases remain to be fully clarified. Agard *et al.* [92], using a quantitative stable isotope labeling with amino acids in cell culture (SILAC) approach in THP-1 cells, identified 82 putative caspase-1-cleaved products and three putative caspase-4 substrates, but no substrates for caspase-5 [92]. Since caspase-1 possesses an extensive range of substrates, it may play a more critical role in inflammatory processes than caspases-4 and -5 in macrophage cells.

Abrahams *et al.* [93,94,95] reported that placental trophoblasts express not only Toll-like receptors (TLRs) but also NLRs. Several NLRs and known activators of DAMPs and PAMPs have recently been identified (Table 1). To date, 14 NLR proteins (NLRPs) have been discovered in addition to NOD proteins, and some receptors are activated by infectious stimuli, such as pathogens in trophoblasts [96]. Among the NLRs, Nuclear factor-kappa B (NF-κB) and mitogen-activated protein kinases possess diverse roles and activate different signaling pathways in inflammation processes [97,98]. Clinical studies have demonstrated a significant correlation between placental infection/inflammation and prematurity [99].

**Table 1:** NLRs-recognized damage-associated molecular pattern DAMP and pathogen-associated molecular patternPAMP.

NLRs	DAMP	PAMP
NOD1		Bacterial peptidoglycan, γ-d-glutamyl-meso-diaminopimelic acid
NOD2		Bacterial peptidoglycan, Muramyl dipeptide
IPAF		Bacterial (Salmonella typhimurium, Pseudomonas aeruginosa, Legionellapneumophila)
NALP1		Bacterial
NALP3	Hyaluronan, uric acid crystals, ATP, silica, asbestos, alum	Bacteria (*Staphylococcus aureus*, *Shigella flexneri*), viruses (Adenovirus, Influenza virus), fungi (*Saccharomyces cerevisiae*), muramyl dipeptide, bacterial pore-forming toxin, bacteria RNA, malarial crystals

The inflammasome plays a vital role in both macrophage immunity and cancer cell progression [8,80]. Wang *et al.* [8] systematically investigated the interactome components of inflammasomes in nasopharyngeal carcinoma using the iTRAQ quantitative assay. Using this method, 172 non-redundant proteins were identified in the inflammasome-interacting complex of HK1/ASC cells. Within the interactome profile, the end-binding protein 1 (EB1) is crucial for stimulating the inflammasome via direct interactions with AIM2 and ASC, both *in vitro* and *in vivo* [8]. EB1 is an adenomatous polyposis coli (APC)-binding protein that regulates microtubule polymerization [100]. Several studies have demonstrated crucial roles of EB1 in biological processes, such as mitosis and signal transduction [101,102]. Furthermore, numerous microtubule-associated proteins are highly expressed during macrophage activation [103]. Based on these findings, microtubule-associated proteins are proposed to play a role in pre-activation or stabilization of the inflammasome [104].

Pre-eclampsia (PE) is a serious disorder that adversely affects ~5% human pregnancies. No useful biomarkers or approaches are currently available to identify and treat pre-eclampsia [105]. Subgroup classification is an effective approach to improve the accuracy of predictions for pregnancy outcomes to individualize breast cancer prognosis and treatment [106]. Clinically, gestational hypertension and proteinuria are the basis of the diagnosis in pre-eclampsia [107]. Cox *et al.* [108] used the shotgun proteomics approach to identify biomarkers of pre-eclampsia with diverse subgroups from plasma membrane proteins at the blood interfaces. In total, 1181 plasma membrane proteins were identified, among which 171 were highly expressed at the maternal blood-trophoblast interface and 192 at the fetal endothelial interface. Various genes from distinct subgroups were enriched. The finding that clinically similar pre-eclampsia patients display distinct gene expression profiles in the placenta implies the existence of divergent molecular mechanisms in pre-eclampsia [108].

A considerable number (12.3%) of live pregnancies are influenced by preterm delivery in the US, which is a main risk of neonatal mortality and morbidity and a considerable burden on the healthcare system [109]. Infection is common and a leading cause of neonatal mortality and morbidity worldwide [110,111]. A significant clinical correlation between infection and preterm labor has been demonstrated, especially for infants delivered before 30 weeks [112]. Several studies have demonstrated that numerous pro-inflammatory genes are up-regulated in both myometrium [113,114] and fetal membrane [115,116] with labor onset. The inflammatory cells enter the uterus and elevate levels of pro-inflammatory cytokines in the parturition process with both preterm and term labor [117]. The expression levels of TNF-α, IL-1β, IL-6 are increased in amniotic fluid [118,119]; furthermore IL-1β and IL-6 expression are elevated in the myometrium [120], amnion [121] and choriodecidua [121]. NF-κB is an inflammatory-associated transcription factor, which is activated in response to infection and pro-inflammatory cytokines induced in labor. Numerous labor-associated and pro-inflammatory genes are influenced by NF-κB, and aberrant NF-κB activity can result in a series of inflammatory-related disorders [122,123]. Moreover, scientists have reported the p65 expression level is elevated in both nuclear and cytosolic fractions in fundal uterine segment myometrium in human labor [124]. Stjernholm-Vladic *et al.* [125] reported that NF-κB expression in the nucleus is increased at parturition in the cervix. Additionally, NF-κB DNA binding activity in the amnion is regulated by IL-1β such as p50/p65 heterodimer, p50 and p65 homodimers [126]. Allport *et al.* [127] reported that the DNA binding and transcriptional activities of NF-κB are elevated in amnion cells following spontaneous labor compared with amnion cells at term, suggesting that in labor, NF-κB possesses a critical role in the amnion. Take together, we speculate that NF-κB signaling pathway play a vital role in placental development, inflammation and the parturition processes both in term, and preterm labor.

Diagnosis of infection in preterm infants is extremely difficult. Clinical diagnosis is based on a number of non-specific characteristics, such as difficulty in breathing, unstable temperature and heart rate, and jaundice [128,129]. Therefore, a rapid and reliable diagnostic test is essential for the clinical identification of neonatal infection. Kingsmore *et al.* [7] used immunoassay arrays to detect 142 cytokines in neonatal serum. Eight serum proteins associated with inflammation, fibrinolysis and coagulation were markedly elevated in infected neonates, including interleukin 18, interleukin 2 soluble receptor α, P- and E-selectins, neutrophil elastase, C-reactive protein, urokinase plasminogen activator and its cognate receptor. Multiplexed immunoassays of the serum biomarker panel have utility for rapid diagnosis and improve the diagnostic performance for neonatal infection [7].

Amniotic fluid (AF) is a complex and dynamic biological fluid that provides nutrients and protection required for fetal growth. Hence, qualitative and quantitative integrity of AF is essential for normal fetal development during gestation. Free diffusion occurs between the fetus and the AF through fetal skin, placenta and the umbilical cord at 10 to 20 weeks gestation [130]. Therefore, analysis of AF components before skin keratinization that occurs between 19 and 20 weeks of gestation may provide critical information for predicting the physiological and pathological status of the fetus. Cho and co-workers [6] investigated the complexity of AF using three differential fractionation approaches to increase coverage, specifically, two-dimensional LC-MS/MS as well as LC-SDS-PAGE-LC-MS/MS platforms. Based on these methods, 842 non-redundant proteins were identified in the AF proteome. Within the AF proteome, biomarkers from several categories have been identified, including intra-amniotic infection, preterm delivery and chromosomal anomalies of the fetus. These findings provide clues that aid in determining the capability of amniotic fluid and detection of significant biomarkers for prenatal diagnosis of fetal abnormalities.

## 7. Conclusions

The thyroid hormone is implicated in various processes, including cancer biology, lipid metabolism, autophagy and inflammation [37,54,131,132]. Inflammation is a critical cellular process in placental development and pregnancy [133]. To date, no useful approaches have been developed to predict pregnancy complications, such as preterm labor. Diagnosis of infection in preterm infants is complex, and clinical identification of neonatal infection is a difficult process based on nonspecific features, such as jaundice, difficulty in breathing and feeding, and unstable temperature [128,134]. However, several cytokines and chemokines are significantly altered in infected neonates [7]. Furthermore, thyroid hormone plays a role in inflammation, placental development and pregnancy-related diseases [70,72]. The inflammasome is clearly implicated in placental trophoblast activity and pregnancy complications [96,135]. In summary, we have provided an overview of several proteomic approaches that may be effectively applied to identify useful biomarkers for the prediction of pregnancy-related diseases and establish specific roles of the thyroid hormone and inflammasome in placenta and pregnancy disorders.
